# Epidemiologic study of traffic accidents and determining the status of injured and their therapeutic benefits Attendees in Zanjan province trauma center in 1397 

**Published:** 2019-08

**Authors:** Kimia Eliasi, Majid Rezvanifard, Soghree Panahi

**Affiliations:** ^*a*^Bachelor of Disaster Management, Zanjan University of Medical Sciences, Ayatollah Mousavi Hospital, and Quality Improvement Office.; ^*b*^Department of Health, Zanjan University of Medical Sciences, Zanjan Iran.; ^*c*^Nursing expert, Ayatollah Mousavi Hospital, Quality Improvement Office.

## Abstract

**Background:**

Traffic accidents have claimed about one million people worldwide and injures several million people. And in the meantime Iran as one of the countries has most accidents and deaths are reported. This study was conducted with the aim of investigating the epidemiology of traffic accidents in Zanjan province in 1397.

**Methods:**

The present study is a descriptive-analytic type that involves using national and global data WHO reports on driving accidents, Reports from the Iranian Ministry of Health - Disaster Management Center, Red Crescent Center and statistical data of the provincial trauma center in the population covered by Zanjan Which has been traumatized in 1397 and the traffic indicators of the province are compared with a number of provinces and neighboring countries.

**Results:**

The density and per capita route in Iran is lower than in developed countries. Of the total accidents in the province, 38.9% were accidents and 61% accidents inside the city. Most of the accidents were 64.5% in the main ways and were the most male victims and the average age was between 45-18 years. And most injuries have been due to head injury.

**Conclusions:**

Since most injuries and traffic accidents are related to men and in the age group of 18 to 45 years, therefore, the implementation of politics and organization in the prevention of accidents and accidents, this coral is a key health and safety priority for individuals At the provincial level.

**Keywords:**

Epidemiology, Disasters and Accidents, Traffic

